# Supplementary material for: Photo-Thermally Tunable Photon-Pair Generation in Dielectric Metasurfaces
Source: ACS Nano. 2026 Jan 30;20(5):4079–87. doi: 10.1021/acsnano.5c14740 (PMC12895560; doi:10.1021/acsnano.5c14740)
Supplement: Supplementary file 1 [file nn5c14740_si_001.pdf]

Supporting Information

Photo-Thermally Tunable Photon-Pair Generation in Dielectric Metasurfaces

Omer Can Karaman[*1], Hua Li[†2,3], Elif Nur Dayi[1], Christophe Galland[‡2] and Giulia Tagliabue[§1]

[1]Laboratory of Nanoscience for Energy Technologies (LNET), STI, École Polytechnique Fédérale de Lausanne, 1015 Lausanne, Switzerland

[2]Institute of Physics and Center for Quantum Science and Engineering, École Polytechnique Fédérale de Lausanne, 1015 Lausanne, Switzerland

[3]State Key Laboratory of Coordination Chemistry, Key Laboratory of Mesoscopic Chemistry of MOE, School of Chemistry and Chemical Engineering, Nanjing University, 210023 Nanjing, China

January 13, 2026

[*]Equal contributions

[†]Equal contributions

[‡]Correspondence email address: chris.galland@epfl.ch

[§]Correspondence email address: giulia.tagliabue@epfl.ch

Supporting Note 1: Full-wave simulations of metasurfaces

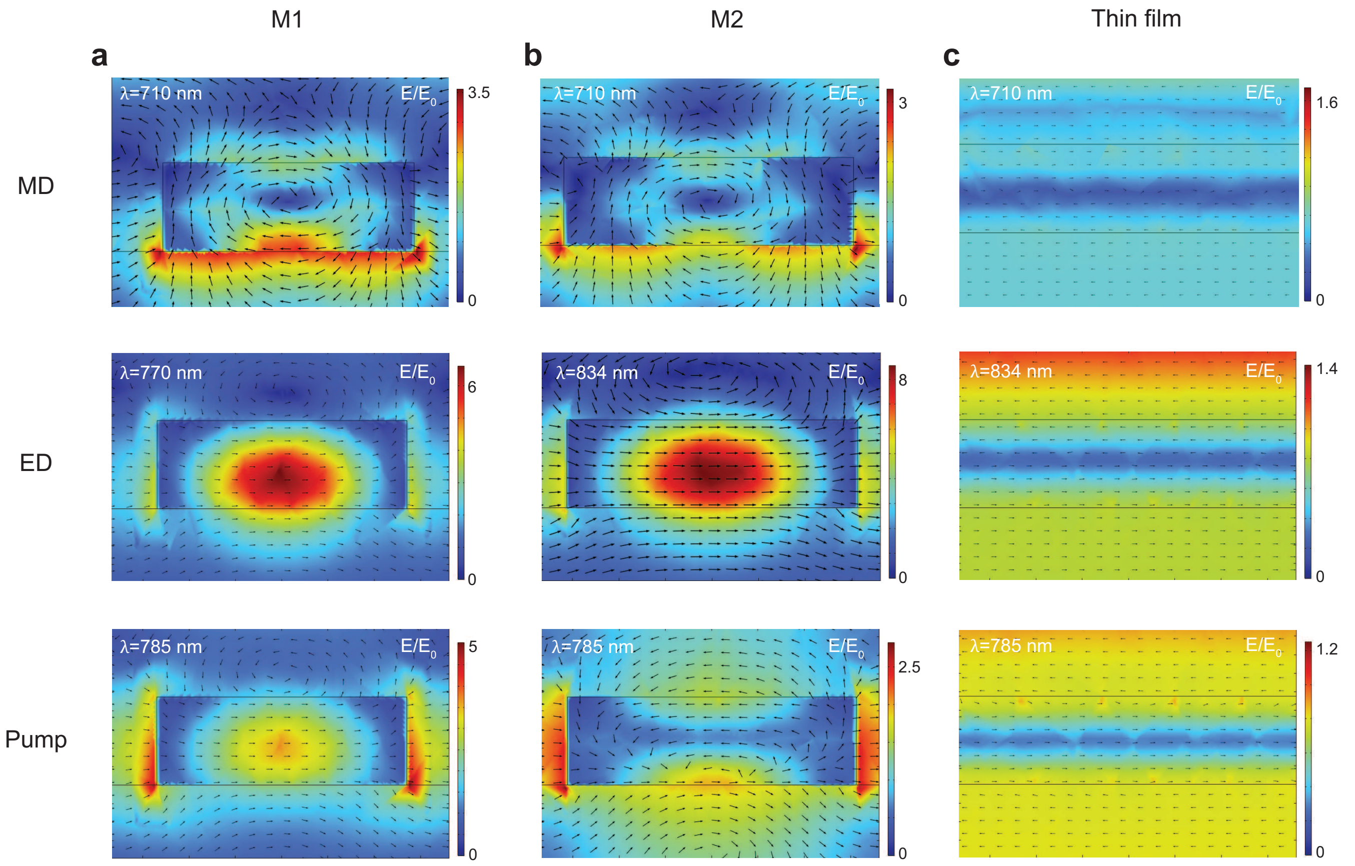

Figure S1: **a–c**, Cross-sectional maps of the normalized electric field amplitudes E/E_0 for metasurface 1 (M1, panel a), metasurface 2 (M2, panel b), and the unpatterned amorphous silicon thin film (panel c). E_0 is the amplitude of the background fields (in vacuum). The top row (MD) corresponds to the magnetic dipole resonance wavelength ($\lambda = 710\,\text{nm}$); the middle row (ED) shows the electric dipole resonances at $\lambda = 770\,\text{nm}$ (M1) and $\lambda = 834\,\text{nm}$ (M2). The bottom row displays the electric field distributions at the pump wavelength used in the experiments ($\lambda = 785\,\text{nm}$). Arrows indicate the in-plane direction of the electric field vectors. Strong localized field enhancements are observed in the metasurfaces, especially near resonance, while the unpatterned thin film exhibits low and rather homogeneous field intensities due to the absence of modal confinement.

Supporting Note 2: Photo-thermo-optical simulations

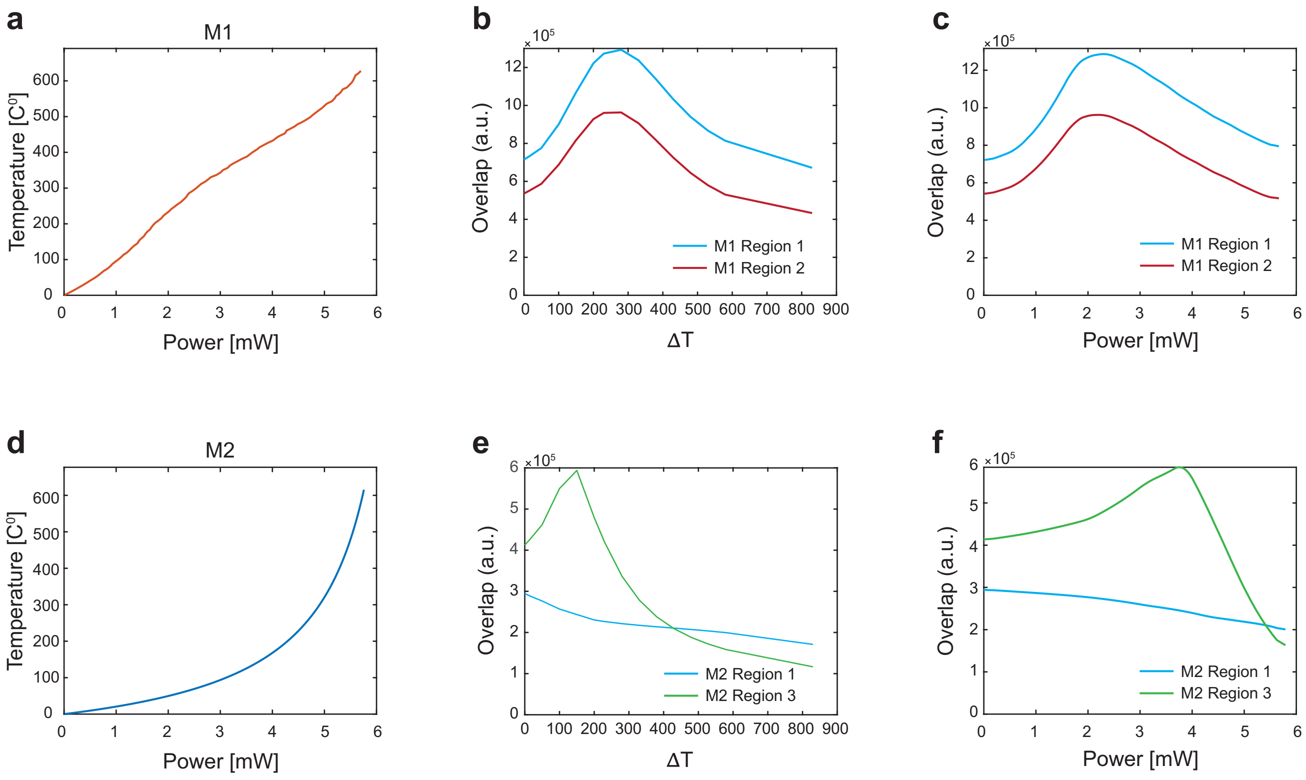

Figure S2: **a,d**, Simulated steady-state temperature increase of M1 and M2 metasurfaces as a function of pump laser power, calculated using an effective thermal capacitance model. **b,e**, Overlap integral magnitude as a function of temperature increase ΔT for detection bands of interest in M1 (detection band 1, detection band 2) and M2 (detection band 1, detection band 3). **c,f**, Power-dependent overlap integral magnitudes derived by combining the thermal response curves from (a,d) with the ΔT-dependent overlap from (b,e). The results indicate a power-tunable nonlinear interaction strength driven by photo-thermal resonance shifts. Distinct power levels yield maximal overlap depending on the detuning of the signal/idler wavelengths with respect to the resonance mode shifts.

To quantitatively understand the influence of thermal effects on the SFWM efficiency in metasurfaces, we computed the steady-state temperature rise and the resulting variation in the nonlinear overlap integral. Figure S2 summarizes the simulation results for metasurfaces M1 and M2. Figures S2a and d show the calculated temperature increase ΔT as a function of pump power for M1 and M2, respectively. These values were obtained using a quasi-CW (continuous wave) heat balance model, assuming an effective thermal capacitance calibrated from absorptance and damage threshold estimates. The high repetition rate (80 MHz) of our pulsed laser ensures

that the metasurfaces experience quasi-steady state heating under experimental conditions.

Figures S2b and e present the computed overlap integral magnitude as a function of ΔT for two SFWM collection windows in each metasurface (M1: detection bands 1 and 2; M2: detection bands 1 and 3). As the temperature increases, the thermo-optic spectral shifts of the resonant modes alter the overlap between the pump, signal, and idler fields. This results in a non-monotonic dependence of the nonlinear overlap on temperature: certain detection bands exhibit a peak in the overlap when the resonance condition optimally aligns with the SFWM emission bands.

Finally, Figs. S2c and f combine the ΔT–power relationship with the temperature-dependent overlap results to yield the power-dependent nonlinear overlap integrals. These power-dependent curves reveal that the effective interaction strength is not simply quadratic in power (a power-independent value is expected for quadratic dependence), but rather exhibits saturation or even decline at high powers, depending on the spectral detuning of the resonance with respect to the SFWM windows. This behavior mirrors the measured photon-pair generation rates in the main text and confirms that resonance-enhanced SFWM in a-Si metasurfaces is tunable via photo-thermal effects.

These simulations validate the observed deviations from ideal quadratic scaling and support our model in which the thermo-optically modulated overlap integral governs the SFWM brightness.

Supporting Note 3: Grain size analysis from X-Ray diffraction patterns

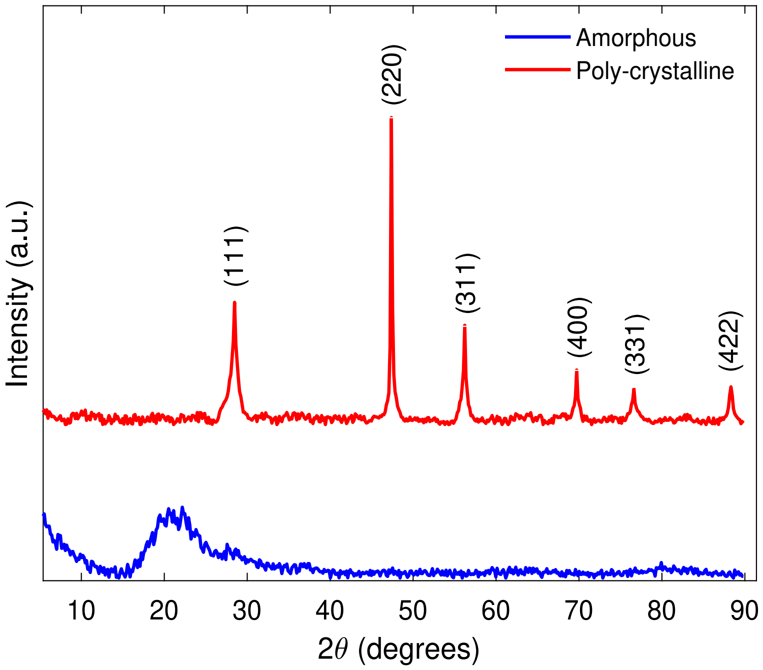

Figure S3: X-ray diffraction (XRD) patterns of amorphous and polycrystalline silicon thin films, acquired using Cu Kα radiation (λ = 1.5406 Å) at a fixed incidence angle of 0.5°. The amorphous silicon sample (blue curve) displays a broad diffuse scattering feature centered near 22°, indicative of a lack of long-range crystalline order. In contrast, the polycrystalline silicon sample (red curve) exhibits sharp Bragg reflections corresponding to the (111), (220), (311), (400), (331), and (422) planes of the diamond cubic silicon structure (space group Fd$\bar{3}$m), confirming the presence of long-range crystallographic order. The pronounced intensity of the (220) peak suggests a possible preferred orientation along this crystallographic direction.

Table 1: Crystallite size estimation using Scherrer equation after instrumental broadening correction.

**Miller Index**	**2θ (deg)**	**$FWHM_{sample}$ (deg)**	**$FWHM_{instr}$ (deg)**	**β (rad)**	**Grain Size (nm)**
(111)	28.451	2.3984	0.2285	0.04167	3.43
(220)	47.392	0.5265	0.2322	0.00825	18.36
(311)	56.204	1.5693	0.2438	0.02706	5.81
(400)	69.721	1.5006	0.2509	0.02582	6.54
(331)	76.611	2.7702	0.2933	0.04808	3.68
(422)	88.319	1.7019	0.2731	0.02932	6.59

Crystallite size was estimated from X-ray diffraction (XRD) peak broadening, with instrumental effects removed using a certified standard, following a standard procedure previously reported[1, 2]. Grazing-incidence XRD (GI-XRD) scans of the polycrystalline silicon thin films were analyzed by fitting each detected diffraction peak with a Voigt-type function using a $\pm 1^\circ$ fitting window around each maximum. The apparent peak broadening (FWHM) was corrected for instrumental contributions using a NIST SRM 660c LaB_6 standard, measured under identical conditions. Peaks in the standard were fitted using Gaussian functions, and the resulting FWHM values were interpolated across 2θ to construct an instrument resolution profile. For each sample peak, the corrected broadening

$$\beta = \sqrt{\beta^2_{\text{sample}} - \beta^2_{\text{instrument}}}$$

was used in the Scherrer equation:

$$D = \frac{K\lambda}{\beta \cos\theta}$$

where $\lambda = 1.5406\,\text{Å}$ (Cu Kα radiation), $K = 0.9$ is the shape factor, and β is the corrected FWHM in radians. Peaks were indexed to the (111), (220), (311), (400), (331), and (422) reflections of cubic silicon (space group Fd$\bar{3}$m). Calculated crystallite sizes ranged from approximately 3 nm to 18 nm, as shown in Table 1, with the largest size observed in the (220) direction, potentially reflecting preferred orientation.

Supporting Note 4: Polarization-resolved photon correlation and brightness in a-Si and poly-Si thin films.

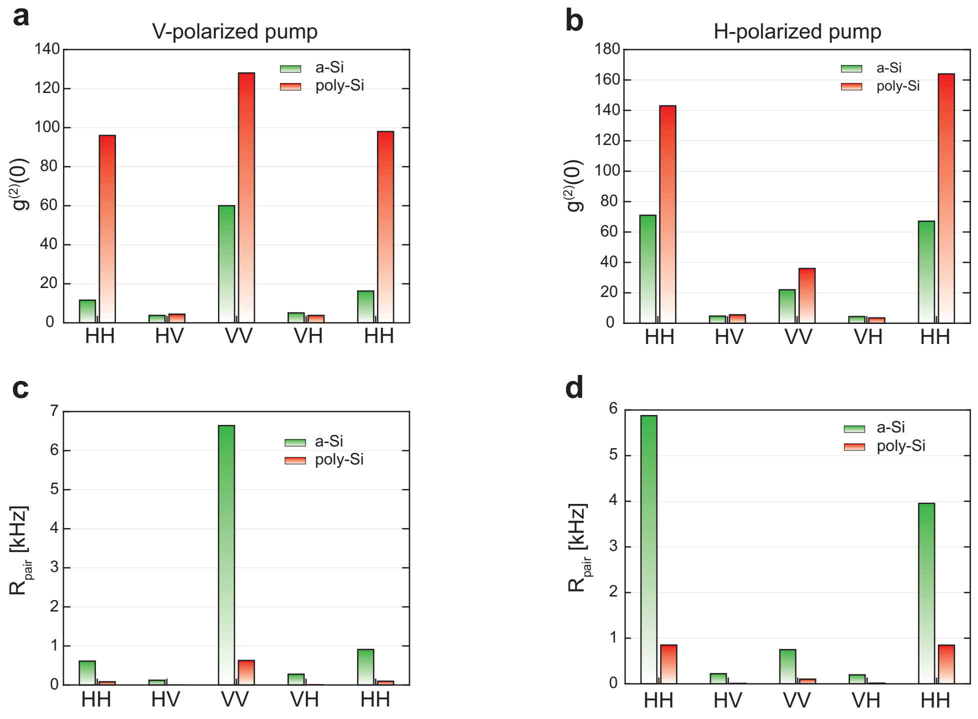

Figure S4: **Polarization-resolved photon correlation and brightness in a-Si and poly-Si thin films.** **a,b** Second-order correlation $g^{(2)}(0)$ of a-Si (green) and poly-Si (red) under V-polarized **(a)** and H-polarized **(b)** pumping at 4.8 mW, for four detection bases (HH, HV, VV, VH). **c,d** Corresponding photon-pair generation rates R_{pair} under V and H-polarized pumping at 4.8 mW, respectively. silicon avalanche photodiodes (SPDs) are filtered to 739–752 nm and 821–837 nm; the polarization basis refers to the signal/idler detection channels.

To compare the polarization dependence of spontaneous four-wave mixing (SFWM) in different silicon phases, we measured photon-pair correlations and generation rates in unpatterned 100 nm-thick amorphous silicon (a-Si) and polycrystalline silicon (poly-Si) thin films. The samples were excited with a femtosecond-pulsed laser at 785 nm (100 fs, 80 MHz) at an average pump power of 4.8 mW. Signal and idler photons were spectrally selected using 10 nm bandpass filters centered at 739–754 nm and 820–835 nm, respectively.

Polarization-resolved measurements were performed in four detection bases: HH, HV, VV, and VH, where the first (second) letter refers to the signal (idler) polarization. Figures S4a,b present the second-order correlation values $g^{(2)}(0)$ for both materials under V-polarized (a) and H-polarized (b) pumping. Poly-Si exhibits the highest $g^{(2)}(0)$ values, reaching up to $\sim$160 in certain co-polarized channels, indicating superior photon pair purity. In contrast, a-Si yields $g^{(2)}(0) \sim 70$ in the highest channel). A possible explanation lies in the different vibrational

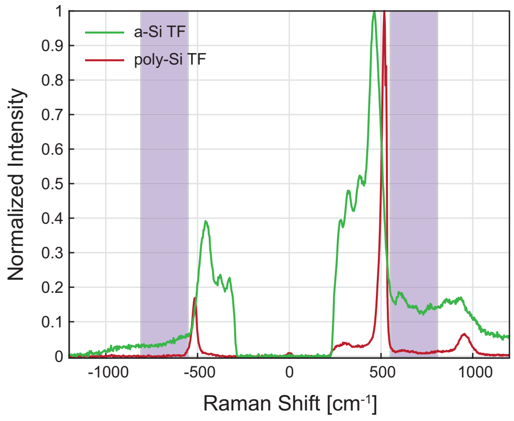

Figure S5: Raman spectra of a-Si and poly-Si thin-films measured with a 488 nm continuous-wave laser at 5 mW power and a beam radius of $\sim$500 nm (corresponding to an excitation intensity of $\sim 6.4 \times 10^5$ W/cm^2). For poly-Si, the dominant first-order transverse optical (TO) phonon peak is observed near 520 cm^{-1}, along with weaker second-order features at $\sim$ 950–1000 cm^{-1} (2TA). For a-Si, the broadened vibrational spectrum comprises the longitudinal acoustic (LA, 312 cm^{-1}), longitudinal optical (LO, 380 cm^{-1}), and transverse optical (TO, 470 cm^{-1}) phonon modes, as well as higher-order modes at 561 cm^{-1} (2LA) and 798 cm^{-1} (2TO) [4]. The dashed region highlights the spectral detection window shown in Figure S4, corresponding to Raman shifts between $\pm$560 and $\pm$792 cm^{-1}.

Raman spectra of these two materials. Due to its disordered structure, a-Si produces a much broader Raman spectrum [3]. As a result, a larger portion of Raman scattered photons overlaps with the detection windows used for SFWM measurements, increasing the overall count rate, but also introducing a higher level of uncorrelated background photons (accidental counts), lowering the $g^{(2)}(0)$ values. In contrast, poly-Si exhibits a narrower Raman peak. This leads to lower collected photon flux and reduced coincidence rates, but also minimizes accidental contributions in the detection bands away from the Raman peaks, resulting in higher observed $g^{(2)}(0)$ values.

The corresponding photon-pair generation rates R_{pair} are shown in **Supporting Information Figure S4c,d**. Here, a-Si consistently outperforms poly-Si, reaching up to 6.5 kHz in the VV channel under V-polarized pumping—over nine times higher than poly-Si under identical conditions. This enhanced brightness arises from a-Si's higher effective third-order susceptibility, estimated from dominant-channel brightness ratios to be $|\chi^{(3)}_{\text{a-Si}}| \approx 3 \times |\chi^{(3)}_{\text{poly-Si}}|$.

Both materials exhibit strong suppression in cross-polarized channels (HV, VH), consistent with the dominance of the $\chi^{(3)}_{xxxx}$ and $\chi^{(3)}_{xxyy}$ tensor elements. The isotropic polarization response of poly-Si is explained by its small crystallite size (3–18 nm, see Supporting Information Note 3), which is much smaller than the optical wavelength. These results underline a material-dependent trade-off: a-Si is advantageous when high photon-pair flux is required, while poly-Si is more suitable for applications demanding maximal photon pair purity.

To clarify the origin of the different photon-pair statistics reported in Figure S4, we measured the Raman spectra of unpatterned a-Si and poly-Si thin films under 488 nm continuous-wave excitation (5 mW, $\sim$500 nm

beam radius). As shown in Figure S5, poly-Si exhibits a sharp first-order TO phonon peak at $\sim 520\ \mathrm{cm}^{-1}$ together with weak second-order features at higher shifts (2TA near 950–1000 cm^{-1}). Importantly, these features lie outside our SFWM detection band, resulting in negligible single-Raman scattering background from poly-Si. In contrast, the a-Si spectrum is strongly broadened due to its disordered structure and contains multiple vibrational contributions, including LA (312 cm^{-1}), LO (380 cm^{-1}), TO (470 cm^{-1}), 2LA (561 cm^{-1}), and 2TO (798 cm^{-1}) modes [4]. Several of these modes, notably the 2LA and 2TO peaks, fall directly within the detection band (± 560 to $\pm 792\ \mathrm{cm}^{-1}$), leading to significantly enhanced single-Raman scattering in a-Si. This explains why a-Si exhibits higher accidental counts and reduced $g^{(2)}(0)$ values compared to poly-Si, despite its superior nonlinear response.

Supporting Note 5: $g^{(2)}(0)$ in Metasurfaces and thin film

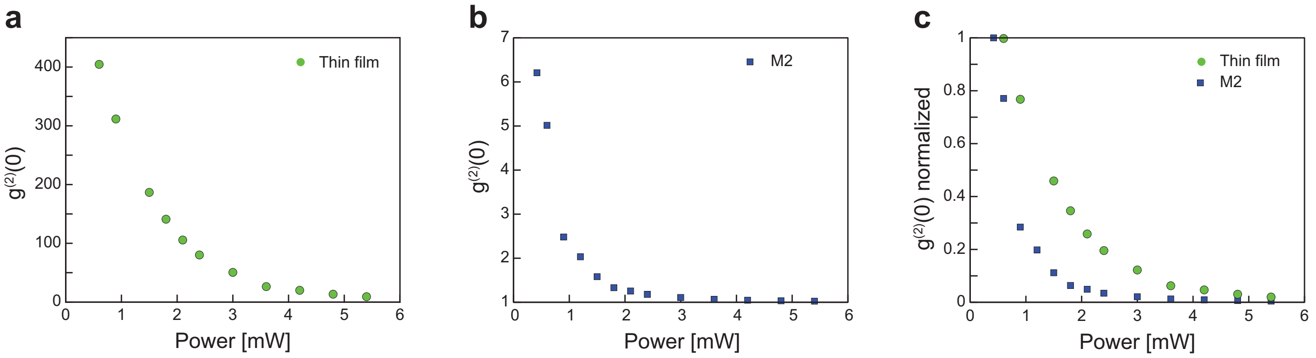

Figure S6: **Power-dependent second-order correlations in thin film and metasurface M2.** (a) Measured $g^{(2)}(0)$ of the a-Si thin film and (b) metasurface M2 as a function of pump power. (c) Comparison after normalizing each dataset to its own dynamic range, showing that $g^{(2)}(0)$ decreases significantly more rapidly in the metasurface.

To clarify why the metasurfaces exhibit a lower and more rapidly decreasing second-order correlation $g^{(2)}(0)$ compared to the unpatterned a-Si thin film, we examine the structure of the accidental coincidences and the role of resonantly enhanced electric fields at the relevant wavelengths. In our experiment, accidental coincidences arise from [5]

$$N_{\text{acc}} = (N_{\text{FWM,i}} + N_{\text{S}} + N_{\text{dark}})\,(N_{\text{FWM,s}} + N_{\text{AS}} + N_{\text{dark}})\,. \tag{S1}$$

which expands into nine distinct contributions. These include pure Raman terms $N_{\text{S}}N_{\text{AS}}$, pure SFWM leakage terms $N_{\text{FWM,i}}N_{\text{FWM,s}}$, and several mixed nonlinear–Raman terms such as $N_{\text{FWM,i}}N_{\text{S}}$ and $N_{\text{FWM,s}}N_{\text{AS}}$. Each of these quantities depends on the internal electric fields evaluated at the pump, signal, idler, Stokes, and anti-Stokes wavelengths [6, 7]:

$$N_{\text{FWM}} \propto |E_{\text{p}}|^4|E_{\text{s}}|^2|E_{\text{i}}|^2, \qquad N_{\text{S}} \propto |E_{\text{p}}|^2|E_{\text{S}}|^2, \qquad N_{\text{AS}} \propto |E_{\text{p}}|^2|E_{\text{AS}}|^2.$$

where E_{S} and E_{AS} are the electric fields at Stokes and Anti-Stokes energies. The true coincidence rate is proportional to the nonlinear overlap integral

$$\Gamma_{s,i} \propto \left| \int \chi^{(3)}\, E_p E_p E_{\text{s}}^* E_{\text{i}}^*\, d^3r \right|^2 \propto |E_p|^4|E_s|^2|E_i|^2. \tag{S2}$$

whereas the mixed accidental terms, such as $N_{\text{FWM,i}}N_{\text{AS}}$, scale as

$$N_{\text{FWM,i}}N_{\text{AS}} \propto |E_{\text{s}}|^2|E_{\text{i}}|^2|E_{\text{S}}|^2|E_p|^6 \propto |E_{\text{s}}|^2|E_{\text{i}}|^4|E_p|^6. \tag{S3}$$

where $|E_{\text{i}}| = |E_{\text{S}}|$ since the idler and Stokes detection bands are the same. Therefore, even when the SFWM rate is enhanced, certain accidental contributions increase more rapidly with pump power than the true coincidences.

The metasurfaces support magnetic and electric dipole Mie resonances (Figure 2a of the main text), which lead to strong enhancement of the internal fields at the pump, signal, and idler wavelengths. These resonances

simultaneously increase the Raman background collected in the same detection bands as the SFWM photons (Figures 2b and S5) and amplify the FWM leakage terms, thereby boosting all components of N_{acc}, including the higher-order mixed terms. Consequently, N_{acc} grows faster in the metasurfaces than in the unpatterned film, leading to a reduction of $g^{(2)}(0) = (\Gamma_{s,i} + N_{\mathrm{acc}})/N_{\mathrm{acc}}$ with pump power.

This interpretation is directly supported by the experimental slopes: the thin film exhibits an approximate $g^{(2)}(0) \sim P^{-1.3}$ to $P^{-1.8}$ dependence, whereas the metasurfaces show a noticeably steeper decay (Figure S6). Moreover, photo-thermal redshifting of the resonances (Figures 2d–e) further increases the spectral overlap of the Raman sidebands with the resonant modes and modifies the field enhancement at the signal and idler wavelengths. This thermo-optic detuning reinforces the imbalance between the growth rates of $\Gamma_{s,i}$ and N_{acc} at elevated powers, a trend captured both experimentally and in our temperature-dependent overlap integral simulations (Figure S2).

In summary, the metasurfaces display lower $g^{(2)}(0)$ because resonant field enhancement amplifies Raman and SFWM background channels simultaneously, while the structure of the accidental coincidence term causes these background channels to grow with a higher effective power law than the true pair generation rate. As a result, the accidental coincidence level increases more rapidly in metasurfaces than in the thin film, yielding a stronger decrease of the measured $g^{(2)}(0)$ with pump power.

References

1. Hwang, G. C., Blom, D. A., Vogt, T., Lee, J., Choi, H.-J., Shao, S., Ma, Y. & Lee, Y. Pressure-driven phase transitions and reduction of dimensionality in 2D silicon nanosheets. en. *Nature Communications* **9.** Publisher: Nature Publishing Group, 5412 (Dec. 2018).

2. Otis, G., Ejgenberg, M. & Mastai, Y. Solvent-Free Mechanochemical Synthesis of ZnO Nanoparticles by High-Energy Ball Milling of -Zn(OH)2 Crystals. en. *Nanomaterials* **11.** Publisher: Multidisciplinary Digital Publishing Institute, 238 (Jan. 2021).

3. Ahn, K. M., Kang, S. M. & Ahn, B. T. Fabrication of High-Quality Polycrystalline Silicon Film by Crystallization of Amorphous Silicon Film Using AlCl3 Vapor for Thin Film Transistors. en. *Journal of The Electrochemical Society* **158.** Publisher: IOP Publishing, H374 (Feb. 2011).

4. Karaman, O. C., Naidu, G. N., Bowman, A. R., Dayi, E. N. & Tagliabue, G. Decoupling Optical and Thermal Dynamics in Dielectric Metasurfaces for Self-Encoded Photonic Control. en. *Laser & Photonics Reviews* **n/a.** _eprint: https://onlinelibrary.wiley.com/doi/pdf/10.1002/lpor.202501014, e01014.

5. Guo, K., Christensen, E. N., Christensen, J. B., Koefoed, J. G., Bacco, D., Ding, Y., Ou, H. & Rottwitt, K. High coincidence-to-accidental ratio continuous-wave photon-pair generation in a grating-coupled silicon strip waveguide. en. *Applied Physics Express* **10.** Publisher: IOP Publishing, 062801 (May 2017).

6. Bobbs, B. & Warner, C. Raman-resonant four-wave mixing and energy transfer. EN. *JOSA B* **7.** Publisher: Optica Publishing Group, 234–238 (Feb. 1990).

7. Hua, X., Voronine, D. V., Ballmann, C. W., Sinyukov, A. M., Sokolov, A. V. & Scully, M. O. Nature of surface-enhanced coherent Raman scattering. en. *Physical Review A* **89,** 043841 (Apr. 2014).
